# Tiling the Silicon for Added Functionality: PLD Growth of Highly Crystalline STO and PZT on Graphene Oxide-Buffered Silicon Surface

**DOI:** 10.1021/acsami.2c17351

**Published:** 2023-01-18

**Authors:** Zoran Jovanović, Urška Trstenjak, Hsin-Chia Ho, Olena Butsyk, Binbin Chen, Elena Tchernychova, Fedir Borodavka, Gertjan Koster, Jiří Hlinka, Matjaž Spreitzer

**Affiliations:** † Advanced Materials Department, Jožef Stefan Institute, 1000 Ljubljana, Slovenia; ‡ Laboratory of Physics, Vinča Institute of Nuclear Sciences—National Institute of the Republic of Serbia, University of Belgrade, 11351 Belgrade, Serbia; § Department of Dielectrics, Institute of Physics of the Czech Academy of Sciences, 182 00 Prague, Czech Republic; ∥ MESA+ Institute for Nanotechnology, University of Twente, 7522 NB Enschede, The Netherlands; ⊥ Key Laboratory of Polar Materials and Devices (MOE) and Department of Electronics, East China Normal University, 200241 Shanghai, China; # National Institute of Chemistry, 1000 Ljubljana, Slovenia

**Keywords:** PLD deposition, rGO buffer layer, epitaxy, STO pseudo-substrate, added functionality, PZT

## Abstract

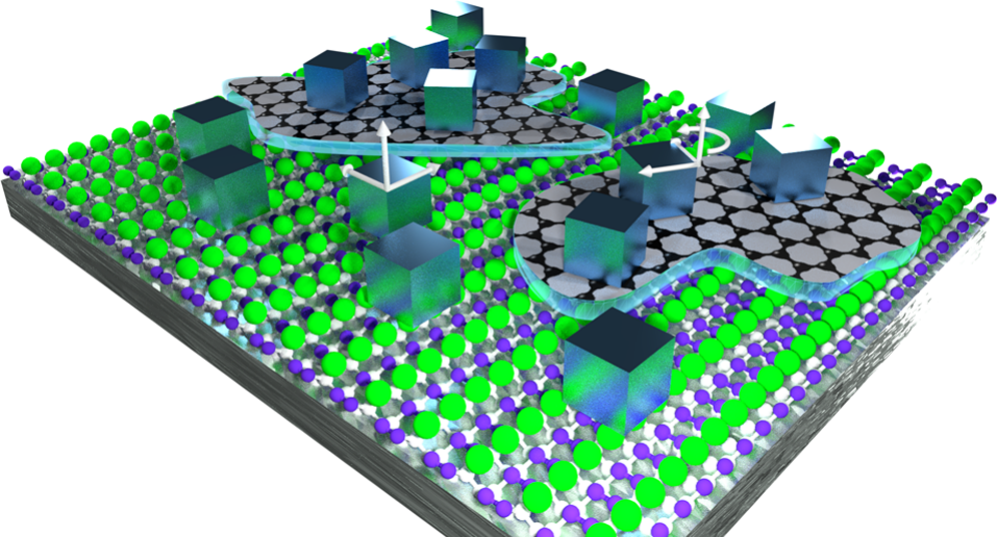

The application of two-dimensional (2D) materials has alleviated a number of challenges of traditional epitaxy and pushed forward the integration of dissimilar materials. Besides acting as a seed layer for van der Waals epitaxy, the 2D materials—being atom(s) thick—have also enabled wetting transparency in which the potential field of the substrate, although partially screened, is still capable of imposing epitaxial overgrowth. One of the crucial steps in this technology is the preservation of the quality of 2D materials during and after their transfer to a substrate of interest. In the present study, we show that by honing the achievements of traditional epitaxy and wet chemistry a hybrid approach can be devised that offers a unique perspective for the integration of functional oxides with a silicon platform. It is based on SrO-assisted deoxidation and controllable coverage of silicon surface with a layer(s) of spin-coated graphene oxide, thus simultaneously allowing both direct and van der Waals epitaxy of SrTiO_3_ (STO). We were able to grow a high-quality STO pseudo-substrate suitable for further overgrowth of functional oxides, such as PbZr_1–*x*_Ti*_x_*O_3_ (PZT). Given that the quality of the films grown on a reduced graphene oxide-buffer layer was almost identical to that obtained on SiC-derived graphene, we believe that this approach may provide new routes for direct and “remote” epitaxy or layer-transfer techniques of dissimilar material systems.

## 1 Introduction

Heteroepitaxy, a case where the deposited layer made of different material has well-defined in- and out-of-plane crystal orientations with respect to the host substrate, has been developed to enable the integration of heterogeneous, high-quality systems for achieving unique functionalities.^1^ Fabrication of heterogeneous layered structures is often challenging, especially when the layer and the substrate possess different intrinsic properties, e.g., large lattice and thermal mismatch between materials would result in a high density of defects. Also, a different chemical reactivity often results in interfacial reactions, which hinders the epitaxial integration of functional oxides, especially in the case of the silicon substrate. Several traditional approaches based on wet chemistry^2^ and high-temperature treatments^3^ are being used for the removal of native oxide from the silicon surface, while state-of-the-art methods use Sr-/SrO-assisted deoxidation under ultrahigh vacuum (UHV) conditions, achieving not only deoxidation but also an atomic-level control of the integration process. Previously, molecular beam epitaxy (MBE)^4−11^ and atomic layer deposition (ALD)^12,13^ methods were the only UHV methods capable of *in situ* deoxidation of Si and formation of a Sr-reconstructed silicon surface. Recently, in the case of the pulsed-laser deposition (PLD) method, we have achieved this using strontium^14−16^ and strontium oxide.^17−19^

The “remote” heteroepitaxy, as an alternative approach to the direct heteroepitaxy, has been recently reported, where a graphene interlayer partially screens the substrate electrostatic potential, reduces the nucleation rate, and simultaneously promotes the growth of the epilayer.^20−22^ It is demonstrated that the amount of energetically favorable dislocations for relaxing the lattice strain, which primarily results from a lattice mismatch between the overgrown layer and the host substrate, can be largely reduced thanks to two-dimensional (2D) materials that alleviate the interaction when the graphene-coated substrate was used for epitaxy.^20,23^ In this regard, epitaxial III–V materials with low-density defects have been successfully grown on top of mono- or bilayer graphene-coated substrates, followed by exfoliation and transfer onto the target substrate to produce high-quality devices.^24,25^ In such cases, the self-organized in-plane ordering of nanostructures has been found to be important for various functional heterostructures.^26^ On the other hand, complex-oxide materials showing extensive functionalities including ferromagnetism, ferroelectricity, and piezoelectricity have also been explored to realize epitaxial growth on graphene-buffered substrates.^22,27^ For example, Kum et al. demonstrated high-quality single-crystalline BaTiO_3_ (BTO) and SrTiO_3_ (STO) epilayers grown on graphene-coated STO substrates.^27^ Dai et al. also reported that the highly oriented BTO film with good piezoelectric properties could be obtained using a graphene-covered SiO_2_/Si substrate.^28^ Lee et al. reported the preparation of a highly oriented STO film with partial epitaxy on top of the graphene substrate.^29^ However, the obtained STO layer was actually polycrystalline in spite of the crystallinity improvement compared to the film obtained without the graphene interlayer.^29^ For the purpose of integrating functional oxides with a silicon platform, we have recently compared different templates for the growth of STO pseudo-substrate on Si, a reduced graphene oxide (rGO) among others.^30^ The present study provides more details on the STO growth mechanism and the role of rGO in this process. Furthermore, the potential of the as-prepared STO layer to be used as a pseudo-substrate was evaluated by overgrowing a PbZr_1–*x*_Ti_*x*_O_3_ (PZT) layer. PZT is one of the most prominent compounds among ferroelectric and piezoelectric materials, owing to its large piezoelectric coefficients, high electromechanical coupling, and reversible remnant polarization,^31^ especially at the morphotropic phase boundary,^32^ which is at the interface between tetragonal and rhombohedral phases.^33^ PZT thin films are attractive for a variety of applications in microelectromechanical systems such as sensors, actuators, and energy harvesters.^34−38^ The piezoelectric response of thin films is drastically reduced compared to that of their bulk counterparts due to clamping by the substrate. When an electric field is applied in the longitudinal direction (across the film thickness), the longitudinal expansion is coupled to a contraction in the transverse direction (parallel to the surface). As the substrate constrains the transverse contraction, the effective longitudinal response is diminished. Several declamping strategies have been proposed, including the use of oxide nanosheet buffer layers. A large piezoelectric response has been achieved by growing PZT thin films with a preferred (100) orientation on glass substrates via a seed layer of crystalline Ca_2_Nb_3_O_10_ oxide nanosheets.^39^

The present study investigated the application of rGO as a buffer/seed layer for the integration of functional oxides with the Si platform. Compared to oxide nanosheets, the GO is more easily processable, especially in aqueous solutions. Furthermore, its surface and structural characteristics are well correlated with the resulting physicochemical properties, thus making the prediction of the final behavior of devices easier. The present study reports SrO-assisted deoxidation of the silicon surface in combination with the graphene (or graphene-like) buffer/seed layer. In regard to the buffer/seed layer, the application of rGO sheets is a novel approach for the growth of oxide thin films on a silicon platform. The obtained results also show quite competitive projections in comparison to CVD-derived graphene, although the structural quality of the initial GO is far behind. Thus, for certain applications, our approach might render the application of CVD graphene and its challenging and time-consuming postprocessing unnecessary.

## 2 Experimental Details

Before GO deposition, the as-received Si wafer (p-type, ρ: 1–10 Ω·cm, Si-Mat, Germany) with a size of 5 × 5 mm^2^ was thoroughly sonicated in acetone, ethanol, and ultrapure water for 15 min each. The Si substrate was then transferred into piranha solution (a 3:1 mixture of concentrated sulfuric acid and 30 wt % of hydrogen peroxide solution), and the solution was heated up to 105 °C for 1 h, followed by spontaneous overnight cooling to room temperature. Subsequently, the Si substrate was removed from the piranha solution and rinsed thoroughly with ultrapure water and ethanol, respectively. On the other hand, the as-received GO suspension (4 mg mL^–1^, Advanced Graphene Products, Poland) was centrifuged at 1000 rpm for 10 min to remove large agglomerated and unexfoliated particles, and the newly obtained suspension was mixed with ultrapure water and ethanol (1:1, vol %). As for GO deposition, the spin-coating approach was adopted,^40^ where the piranha solution-treated Si substrate was spun at 8000 rpm throughout the coating process, while the as-prepared GO suspension was applied onto the Si surface in a dropwise fashion, every ∼40 s, to form a certain coverage of GO sheets (up to 40 μL).

The rGO/Si template was introduced into the PLD chamber (Twente Solid State Technology, Netherlands) and first heated for 3 h at 600 °C in an ultrahigh vacuum to remove surface contaminants. In the second step, the SrO was deposited. After that, a substrate temperature was increased to 760 °C to achieve SiO_2_ deoxidation.^18^ Heating was achieved using an IR laser (λ = 800–820 nm, HighLight FAP 100, Coherent) coupled with an IMPAC IGA 5 pyrometer (LumaSense Technologies, Inc.) with an 85% emissivity constant. This was followed by the depositions of STO, LaNiO_3_ (LNO), and PZT in an oxygen atmosphere. The respective deposition parameters are listed in Table S1, Supporting Information (SI). In the last step, the sample was cooled down in 700 mbar O_2_ at a rate of 10 °C min^–1^. Reflection high-energy electron diffraction (RHEED) images were taken after each deposition step (RHEED gun with differential pumping, STAIB Instruments, Germany, coupled with the kSA 400 RHEED analysis system from *k*-Space Associates, Inc.).

After deposition, the surface morphology of the sample was examined using atomic force microscopy (AFM) in the tapping mode (Veeco Dimension 3100 AFM/MFM system), while the images were processed using WSxM software.^41^ The phase composition, crystal structure, and quality of the samples, as well as the epitaxial relationship between the substrate and the deposited layers were determined by means of X-ray diffractometry (XRD). The θ–2θ patterns, rocking curves (RCs), and azimuthal (Φ) scans were collected using an XRD system (Empyrean, Malvern PANalytical) with Cu Kα_1_ radiation (λ = 1.5406 Å). A double-bounce Ge(220) hybrid monochromator was used on the incident-beam side. The diffracted beam in the θ–2θ scans was captured and analyzed by a PIXcel3D detector operating in 1D mode. The RCs were measured using the 0D operation mode of the detector. The quantity of open channels was optimized to ensure the collection of the entire peaks of interest and avoid overlap with peaks from the remaining layers in the heterostructure.

The cross-sectional samples of interfaces were examined by a JEM-ARM200CF probe Cs-corrected scanning transmission electron microscope (STEM) equipped with a cold field emission (FEG) electron source operated at 80 kV, a JEOL Centurio 100 mm^2^ EDXS detector, JEOL STEM detectors (JEOL, Tokyo, Japan), and a GIFQuantum ER dual-EELS system (GATAN-AMETEK, Plesanton). A cross-sectional transmission electron microscopy (TEM) sample preparation was carried out in a focused ion beam—scanning electron microscope (FIB-SEM) Helios Nanolab 650 (FEI, Netherlands) equipped with a vacuum transfer interlock (Gatan) and energy-dispersive X-ray (EDX) spectrometer X-MAX 50 (Oxford, U.K.).

The room-temperature Raman spectra were collected using an RM1000 Micro-Raman spectrometer (RENISHAW) and In-Via Reflex Raman microscope (RENISHAW) in the range of 10–3500 cm^–1^. An RM1000 Micro-Raman spectrometer was equipped with Bragg filters and an argon ion laser operating at 514.5 nm. An In-Via Reflex Raman microscope was equipped with EDGE filters and a laser operating at a wavelength of 488 nm. Both spectrometers were equipped with a 2400 L mm^–1^ diffraction grating and a 50× optical objective. Raman spectra were acquired with six accumulations of 10 s each. The measurements were performed in a backscattering geometry.

The thermal analysis of bulk graphene oxide nanosheets was studied by thermogravimetry and differential thermal analysis (TG-DTA, NETZSCH, Germany) using the Jupiter 449 simultaneous thermal analysis (STA) instrument in the temperature range of 35–1200 °C with a temperature ramp rate of 5 °C min^–1^ in both heating and cooling under an argon environment (purity of 99.99%) with a constant flow rate of 40 mL min^–1^.

Au top electrodes with a diameter of 600 μm were sputtered onto the PZT to fabricate capacitor structures required to measure the electrical properties. The electric field tunability of the dielectric permittivity was measured using an Agilent 16065C bias tee, a DC power source, and a 4284A Precision LCR Meter (Agilent) at a frequency of 1 kHz and an AC amplitude of 100 mV. Polarization versus electric field (*P*–*E*) hysteresis loops were measured using a ferroelectric test system (Precision LC Materials Analyzer, Radiant Technologies) with a series of double triangular bipolar waveforms at a scan frequency of 10 Hz and an amplitude of 10 V. The longitudinal effective piezoelectric coefficient (*d*_33,f_) of the PZT films was determined using a double-beam laser interferometer (aixDBLI, aixACCT, Germany) at a measurement frequency of 1 kHz and an AC amplitude of 500 mV.

## 3 Results and Discussion

### 3.1 PLD Growth of STO on rGO-Buffered Silicon Surface

**Figure 1:**
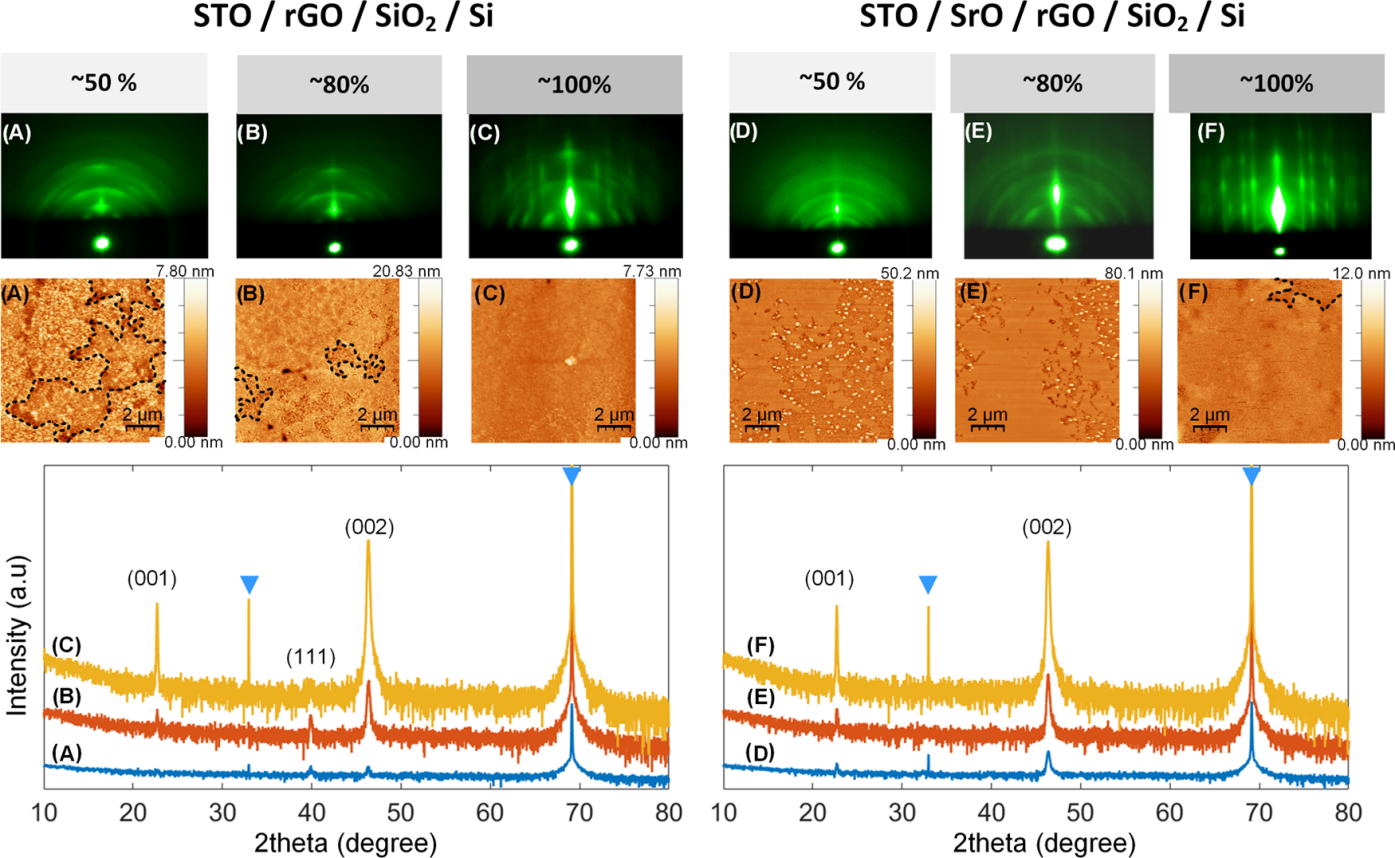
STO grown on the rGO-buffered SiO_2_/Si surface, as seen by RHEED (top), AFM (middle), and XRD (bottom) methods. The rGO coverage of the SiO_2_/Si surface in (A, D), (B, E), and (C, F) cases is ∼50, ∼80, and ∼100%, respectively. In sections (A–C), the STO is grown directly on rGO (and the bare SiO_2_/Si surface), while in (D–F) cases, prior to STO growth, a small amount of SrO, optimal for the deoxidation of silicon surface, was used. The diffractograms are offset for clarity; the triangle denotes diffraction maxima of the silicon substrate. In some of the AFM images, for clarity, a dashed line encircles the regions of STO grown on bare SiO_2_/Si.

Prior to STO deposition, to minimize surface contaminants, the SiO_2_/Si surface with different coverages of GO layers (∼50, ∼80, and ∼100% coverages; Figure S1, SI) was annealed for 3 h at 600 °C in an ultrahigh vacuum. During this process, besides degassing of the sample and sample holder, which mostly includes the desorption of water and volatile hydrocarbons, the GO undergoes a thermally induced reduction (Figure S2, SI), during which a number of functional groups are removed from both edge and basal planes of GO.^42^ Consequently, GO is being transformed into its partially reduced form, rGO. The PLD growth of STO on SiO_2_/Si with different surface coverages of rGO buffer was systematically studied *in situ* and *ex situ*, and the results after the growth of an ∼90 nm STO layer are shown in Figure 1. Prior to STO deposition, the RHEED images from rGO sheets show diffuse patterns arising from the crystalline graphene lattice (rGO part in Figure S3, SI). As the first few STO pulses arrive at the substrate, the RHEED patterns change drastically (∼2 nm, Figure S3, SI) and afterward retain a similar picture until the end of deposition. Characteristic features of the growth are discussed in detail in Figure S3, SI.

The top panel of Figure 1A–C, when ∼90 nm of STO is directly grown on rGO, shows a stark difference in RHEED patterns as the rGO coverage changes from ∼50 to ∼100%. Identifiable streaks along with broken rings obtained in ∼100% of the rGO-covered sample (Figure 1C, top panel) suggest the coexistence of a two-dimensional flat crystalline layer and a textured surface, while in the other two samples with lower rGO coverage (Figure 1A,B, top panel), the crystallinity is relatively poor featuring a series of rings and elongated spots. AFM images (Figure 1A–C, middle panel) show an evident contrast between the regions where STO was deposited on rGO and where STO was directly contacted with silicon (encircled with dashed lines), especially in ∼50 and ∼80% covered samples. The STO layer on ∼100% of the rGO-covered sample exhibits a relatively smooth surface with an RMS value of ∼0.34 nm (∼1 nm for ∼50% sample and ∼1.8 nm for ∼80% sample). From the XRD results (Figure 1A–C, bottom panel), it can be clearly seen that the crystalline quality of STO is greatly improved as the rGO coverage increases, thus indicating the importance of rGO nanosheets in dictating the growth of STO in the preferred orientation. The presence of the (111) peak implies that even if the whole silicon surface is covered with rGO, it is difficult to prepare a single-oriented STO layer, and the presence of this undesirable orientation can be noticed in the RHEED patterns as well.

### 3.2 PLD Growth of STO on rGO-Buffered Silicon Surface after SrO-Assisted Deoxidation

Another set of samples was prepared with a small amount of SrO deposited prior to STO, to assist in the removal of native oxide on regions of bare SiO_2_/Si.^18^ The results after growing ∼90 nm of STO on the SrO-deoxidized Si surface are shown in Figure 1D–F. The coexistence of streaks and broken rings in RHEED patterns are observed in ∼50 and ∼80% rGO-covered samples (Figure 1D,E, top panel), respectively, similar to those prepared without SrO-assisted deoxidation. One should note, however, that the streaky line became narrower and sharper compared to the sample prepared without SrO, indicative of the improved crystallinity of the overgrown STO layer. The improvement of the crystallinity can also be compared in ∼80% rGO-covered samples with and without the SrO layer (Figure 1B,E, top panel) where numerous faint streaks appear as SrO is used. More importantly, a well-defined streaky pattern is obtained in an ∼100% rGO-covered sample (Figure 1F, top panel), indicative of two-dimensional layer growth. Characteristic features of the growth are discussed in detail in Figure S4, SI. AFM results (Figure 1D–F, middle panel) show that while the granular particles are easily formed on the regions where SiO_2_/Si is in direct contact with SrO/STO, the areas buffered by rGO lead to a very smooth STO surface. This is most noticeable in the ∼100% rGO-covered sample with an RMS value of ∼0.8 nm, which can also be inferred from the RHEED image.

XRD results are shown in the bottom panels in Figure 1D–F. In contrast to the samples where STO is grown directly on rGO, this time no (111) orientation is detected irrespective of the rGO coverage if SrO-assisted deoxidation is used. Furthermore, the level of epitaxy is significantly improved as the surface coverage of rGO is increased from ∼50 to ∼100% (with the enhanced intensity of the preferred orientations (001) and (002)). It clearly reveals that a high-quality single-crystal epitaxial STO layer can be realized by benefiting from full surface coverage of the rGO-buffered layer as well as the optimal SrO deoxidation of the Si surface. Also, it was established that 700 °C is the optimal temperature for the growth of a high-crystalline quality STO to be used as a pseudo-substrate (Figure S5, SI).

### 3.3 Crystalline Quality of STO Grown on rGO-Buffered SiO_2_/Si Surface

**Figure 2:**
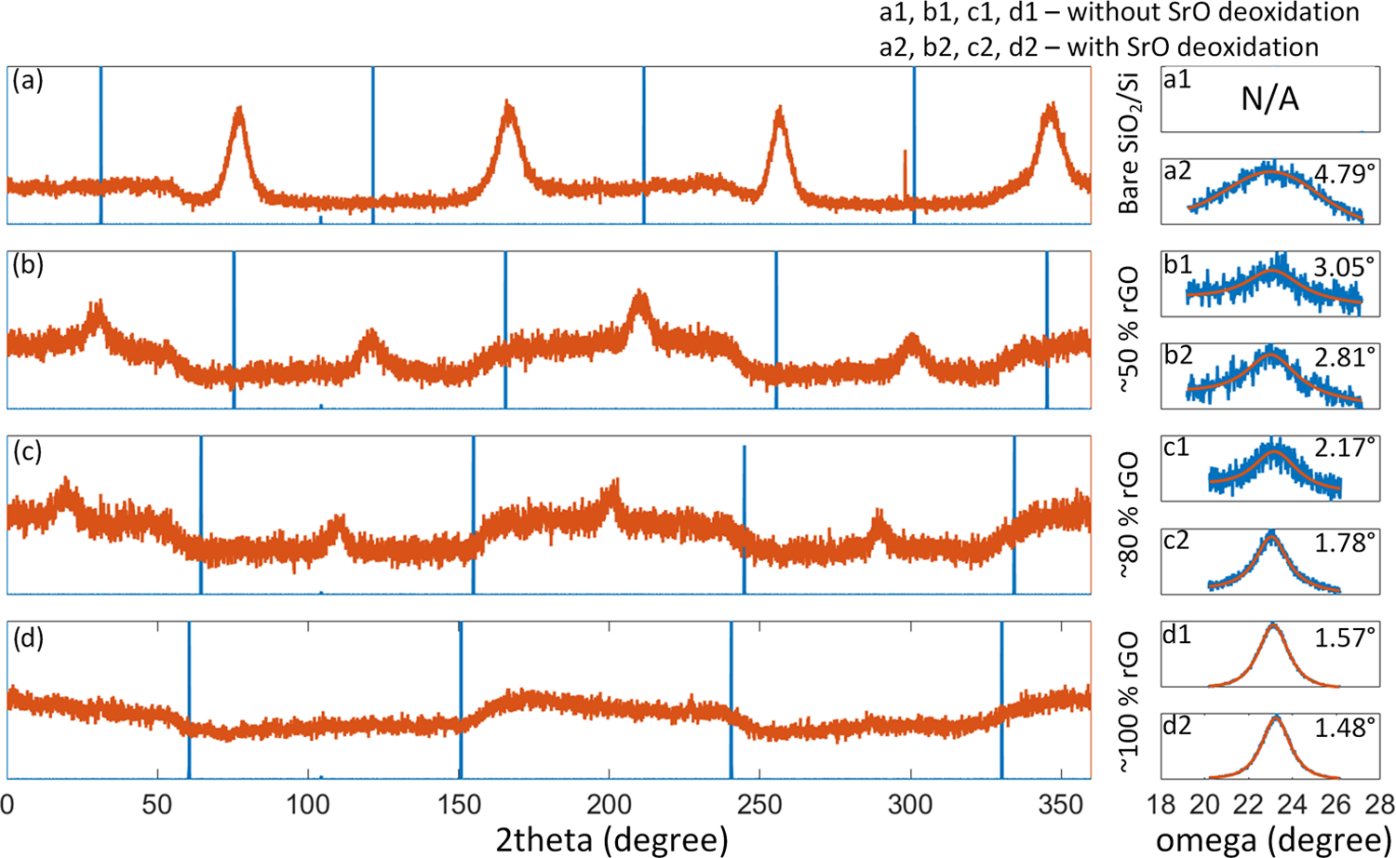
Phi-scan of STO (orange traces, {110} asymmetric set of planes) grown on the rGO-buffered SiO_2_/Si surface (blue lines) after the application of SrO as a deoxidizing agent: (a) bare silicon surface (i.e., 0%): (b) ∼50%, (c) ∼80%, and (d) ∼100% rGO coverages. On the right, the rocking curve analysis of the STO (002) diffraction maxima for the rGO-buffered SiO_2_/Si surface with 0, ∼50, ∼80, and ∼100% rGO coverages: (a1, b1, c1, d1) without SrO and (a2, b2, c2, d2) with SrO-assisted deoxidation. The full width at half-maximum (FWHM) value of each peak is shown in the top-right corner of rocking curve sections.

The XRD azimuthal (Φ) scans of the {110} asymmetric set of planes for the STO films grown on the SiO_2_/Si substrate with different rGO coverages after SrO-assisted deoxidation are shown in Figure 2.

The sample without rGO (panel a) presents a clear 4-fold symmetry. As the rGO coverages increase, the in-plane epitaxial relationship with the Si substrate gets weaker. When the rGO coverage reaches 100%, the in-plane relationship fully vanishes. The reason behind this is that the rGO sheets act as a barrier for the SrO because of which deoxidation reaction was not possible, i.e., removal of native SiO_2_ was hindered. Hence, at locations where the SiO_2_ interlayer is still present, an epitaxial relationship with the Si substrate cannot be achieved (i.e., rGO acts as a seed layer for the STO). The STO sample grown on bare Si but without SrO-assisted deoxidation has shown the same signal shape in panel d (not shown). It was determined that the periodic variation of intensity that is most noticeable in panel d is a consequence of the quadratic shape of the 5 × 5 mm^2^ Si substrate.

The panels on the right-hand side of Figure 2 reveal that there is also a dependence of the crystalline quality i.e., “mosaicity” on the rGO coverage. As the rGO coverage increased, a stark improvement in the crystalline quality, reflected in a lower FWHM value, is observed for both sets of samples—without (panels a1, b1, c1, and d1) and with (panels a2, b2, c2, and d2) SrO-assisted deoxidation. While the in-plane epitaxial relationship with the Si substrate is lost as the surface is completely covered with rGO (panel d), the rGO itself promotes the out-of-plane epitaxial growth of STO (in [001]). Nonetheless, it can be concluded that the SrO contribution to STO epitaxy is more pronounced in the case of samples with lower rGO coverage. The FWHM value obtained in the case of the sample with 100% of rGO coverage is comparable to state-of-the-art PLD-grown STO films on the Si substrate.^43^ The SrO deoxidation contributes to the improvement of the FWHM value, as shown in Figure 2 (b1 vs b2, c1 vs c2 and d1 vs d2). There is no RC in panel a1 due to a complete absence of epitaxy, i.e., no (002) STO peak was present in the case of STO grown on bare Si without SrO-assisted deoxidation (Figure S6, SI).

### 3.4 Implications of the STO Growth on Interface: rGO-Buffered vs Bare SiO_2_/Si Surface

**Figure 3:**
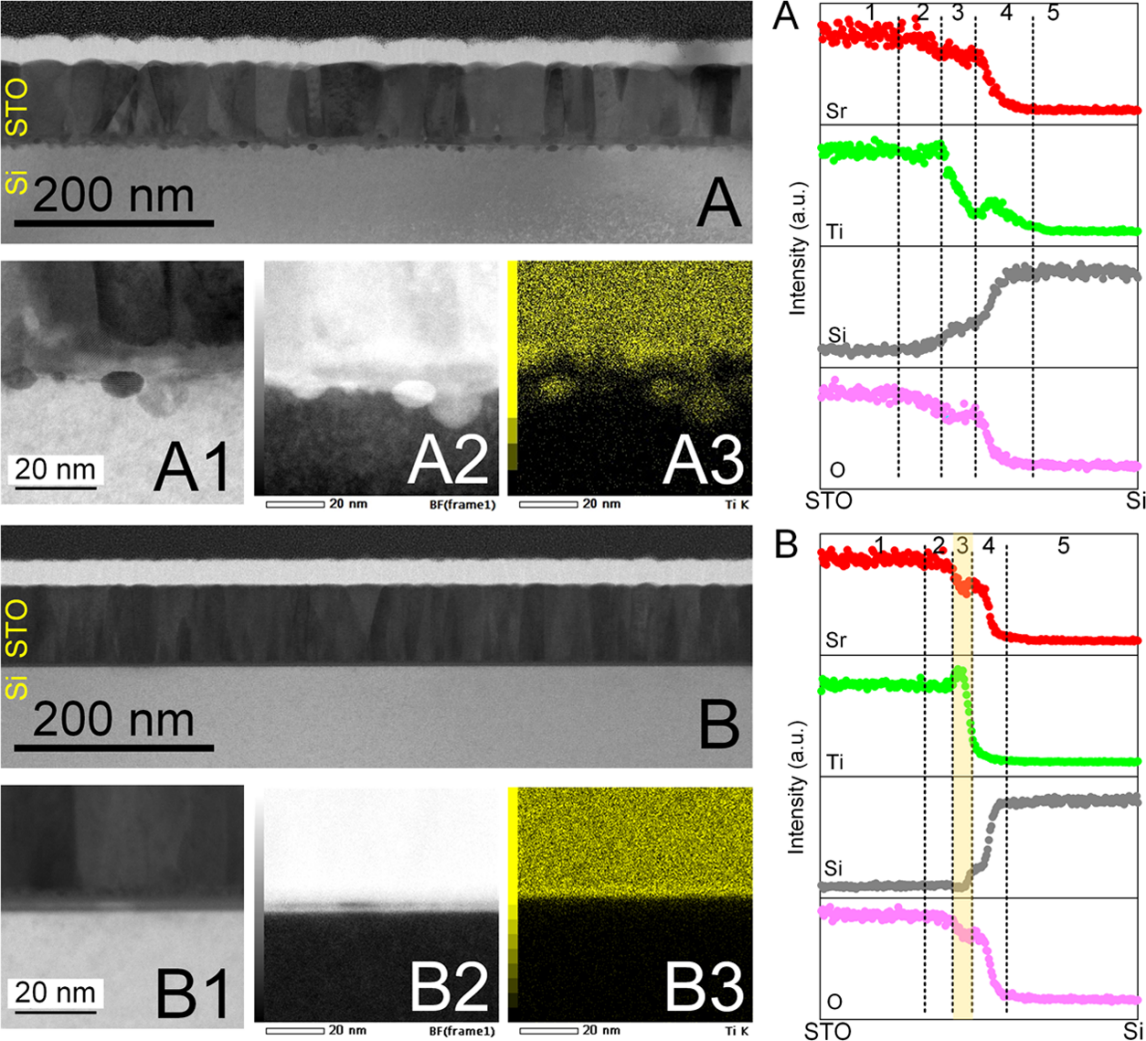
Interface after the growth of 85 nm thick STO layer on (A) bare and (B) rGO-buffered SiO_2_/Si surface. The STO was grown after SrO-assisted deoxidation. (A1, B1), (A2, B2), and (A3, B3) represent the dark-field, bright-field, and Ti-mapping obtained by TEM analysis, respectively. Panels on the right show the elemental distribution of O, Si, Ti, and Sr across the interface after the growth of STO on (A) bare and (B) rGO-buffered SiO_2_/Si surfaces. The respective number denotes regions in the bulk of the STO film (1), interface to the STO side (2), main interface layer where the reaction takes place (3), interface to the Si side (4), and bulk of Si (5). rGO sheets are positioned at region 3 of the rGO-buffered sample, while SrO is situated at region 2 of both samples.

To investigate the STO–substrate interface, a cross-sectional specimen for TEM was prepared by means of the focused ion beam (FIB) technique. The first FIB lamella was cut out of the bare SiO_2_/Si substrate (Figure 3A), while the second one was cut out from the rGO-buffered region (Figure 3B). Both films have similar thicknesses of about 85 nm, measured from the top end of the film to the beginning of the silicon substrate. The interfacial features of both regions that can already be seen in smaller-magnification bright-field transmission electron microscopy (BF-TEM) images, however, differ significantly. The unbuffered region exhibits a nonuniform film/substrate interface, where some particle-like precipitates can be seen protruding into the Si substrate (Figure 3A). The STEM–EDX line profile shown in the right-hand panel (see also full STEM–EDX maps in Figure S7, SI) reveals a slight drop in the Sr and O amounts with the simultaneous increase of Ti and Si at region 2, where the interfacial reaction layer starts. Further, in the interfacial region 3, the drop of the Ti amount is accompanied by the further increase of the Si with the Sr amount staying steady. This implies the formation of a strontium silicide layer in that region (presumably resulting from SrO-assisted deoxidation). In region 4, peaking in the Ti composition line profile with a simultaneous further increase of Si and a drop of Sr and O signals indicates the formation of the titanium silicide. The position of the titanium silicide layer corresponds to the position of the particle-like protrusions at the interface. Although some of the particles were completely crystalline, it was not possible to determine the exact form of the silicide due to the STEM scanning errors and underlying thick Si matrix.

The rGO-buffered region, on the other hand, exhibits a completely flat interface (Figure 3, right, panel B). In the interfacial regions 2 and 3 of the STEM–EDX line profile, an increase in Ti along with a decrease in Sr and O can be seen. In this rGO-buffered area of the STO film, a stronger accumulation of Ti can be observed, but it seems that rGO acts as a barrier for its further diffusion toward the Si substrate. Compared to this, a slightly higher concentration of Sr can be noticed below the rGO. Hence, the interface is much sharper (the thickness of the interfacial region, 2–3–4, drops from ∼35 to ∼12 nm) and there are no particle-like protrusions. It has been documented that the graphene-based material with a lower diffusion energy barrier can facilitate the migration and arrangement of arriving adatoms on the graphene surface during the deposition, whereas the adatoms are more likely to desorb from the SiO_2_ surface because of the relatively higher diffusion energy barrier, which could partly explain the resulting interface between the grown STO films and two different substrates.^44,45^ Therefore, we can conclude that rGO acts as an efficient diffusion barrier, enabling the formation of sharper interfaces and higher-quality epitaxial layer overgrowth. Noteworthily, both STEM–EDX mapping and electron energy loss spectroscopy (EELS) were not able to provide a clear indication of the presence of rGO, although its location can be projected based on the abrupt changes of the elemental concentration. It can also be noticed that the overall morphology of the grown STO films, except for the clear difference observed at the STO–substrate interfaces between the rGO-buffered and bare SiO_2_/Si, appears quite similar in both cases.

Some assumptions related to the STO growth mechanism and interplay between rGO and SrO can be derived from RHEED (Figures S3 and S4) and TEM/EDS (Figures 3 and S7) results. As can be noticed, without SrO deoxidation, the vague STO streaks are visible after the deposition of ∼2 nm STO (Figure S3; 100% rGO coverage). On the other hand, with SrO deoxidation, the STO streaks are more intense and visible already at ∼1 nm (Figure S4; 100% rGO coverage). This initial difference in the quality of the STO seed layer is responsible for the quite different quality of the resulting STO films (see XRD and RHEED in Figure 1).

It is known that STO growth on graphene is facilitated by a good epitaxial registry (<1% mismatch).^46^ So, why SrO deoxidation enhances the STO growth on the rGO-covered silicon surface? On a bare silicon surface, the STO growth is enhanced by the removal of native oxide and Sr-passivation of the uppermost Si layer.^18^ It is probable that the STO grown in these regions influences the growth of STO on the neighboring rGO by acting laterally as a template. However, on a 100% rGO-covered surface, with a proportionally low surface ratio of bare silicon, this contribution can be considered minimal.

If the Si surface is completely covered with perfect graphene, one can assume that graphene represents an effective barrier for the diffusion of heteroatoms through it. In our case, at an ∼100% coverage, the silicon surface is covered by 2–3 layers of rGO. The overlapping of the rGO sheet (lateral dimensions ∼ 1 μm, on average; Figure S1) and their inherent in-layer defective structure^47^ may create a diffusion pathway for Sr in the early stages of deoxidation. As can be inferred from Figure S7, Sr can permeate the rGO layer and reach SiO_2_/Si. Hence, it can be concluded that the deposited SrO partially remains on top of rGO and other parts of it, diffuses as Sr through the rGO layers, and reaches SiO_2_ to form (nonstoichiometric) silicates. We presume that this might change the “wettability” of the rGO surface in which SrO and Sr effectively enhance the STO layer formation and its crystallinity, thus making rGO a better template for STO growth. Future high-resolution TEM and STM studies might provide new insights that can help to elucidate the mechanism of growth in its early stages.

### 3.5 Implication of the STO Growth on the rGO Buffer Layer

**Figure 4:**
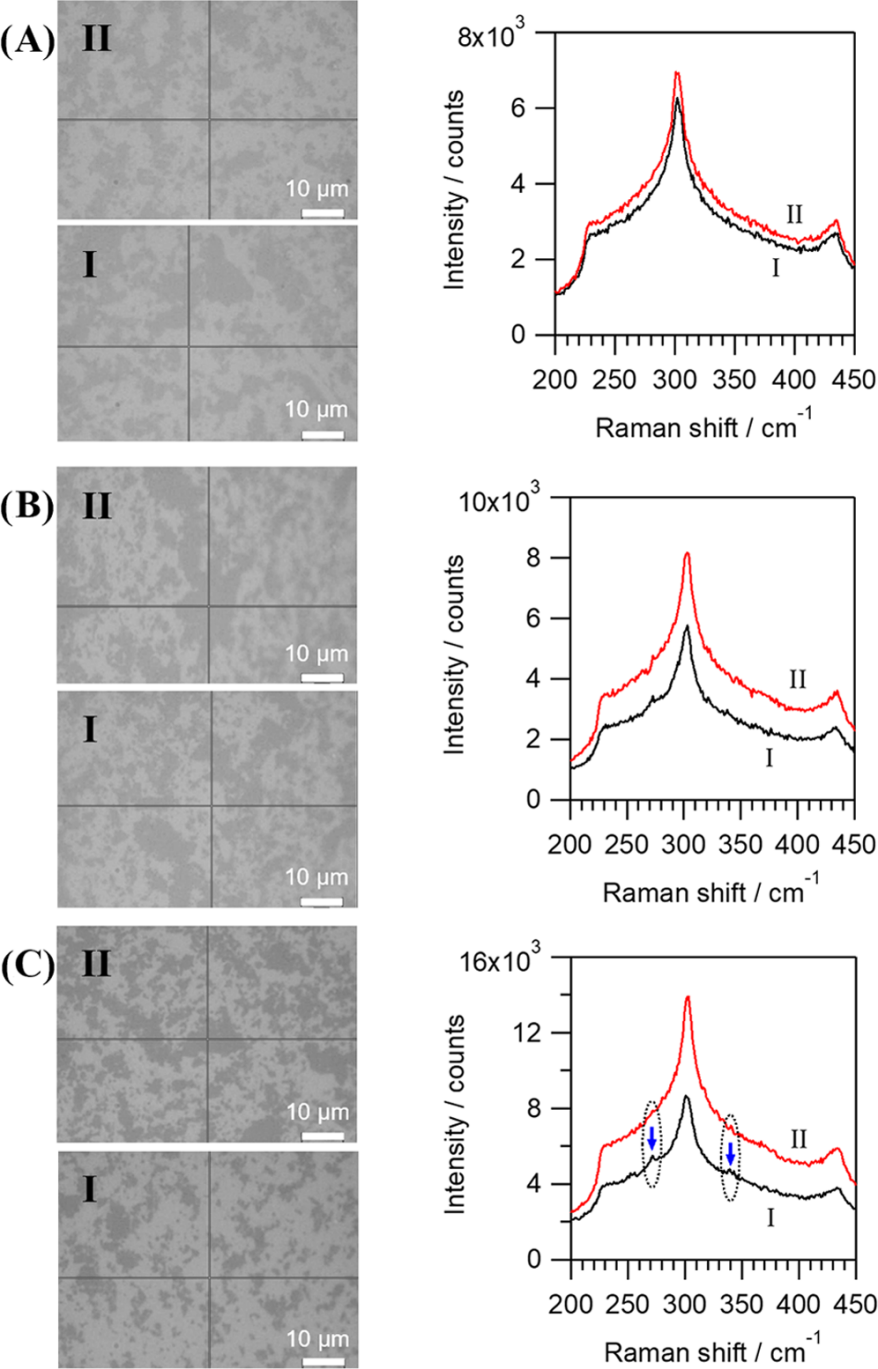
Optical images (left side) and Raman spectra (right side) taken from the samples with a variable thickness of the STO film deposited on a SrO(deox.)/50% rGO-covered/SiO_2_/Si substrate. The thickness of the STO layer grows from top to bottom: (A) 3 nm, (B) 10 nm, (and C) 30 nm of STO. In the optical images of these samples, the rGO-covered regions appear darker compared to that without rGO. The arrows in segment (C) point to the “satellite” peaks (see the main text). The cross-mark of black lines on the photos on the left denotes measurement location.

Raman spectroscopy is a method of choice for the structural characterization of carbon nanomaterials, where the intensity ratio of D and G peaks is often used as an indication of structural quality.^48^ Raman spectra of GO-buffered and bare SiO_2_/Si surface, prior to any treatment, i.e., as-obtained after the spin-coating process, are shown in Figure S8, SI. It can be noticed that the intensity distribution matches an ∼50% GO coverage. In fact, the 50% GO coverage was our starting point for understanding the effects of the STO growth on rGO and Si substrate itself. For this purpose, we have grown on it a layer of 3, 10, and 30 nm thick STOs and performed the Raman analysis (Figure 4).

The left part of Figure 4 shows that the partial coverage by rGO can be inspected optically. It seems likely that the optical contrast between the covered and noncovered areas in the visible range reflected light might be related to the differences in the roughness of the Si/STO surface, evidenced in Figure 1. For the reason given below, we assume that the seemingly darker areas are those covered by rGO. Unfortunately, after the growth of STO, the characteristic D and G Raman lines of rGO could not be well seen neither in the darker nor in the brighter regions. In fact, Raman spectra in both darker and brighter areas do not show any strong first-order Raman lines, except for the 520 cm^–1^ mode of Si. Nevertheless, the spectral region around the characteristic second-order Raman band of Si near 300 cm^–1^ (see the right part of Figure 4) allowed us to make two conclusions. First, we can clearly see that the observed intensity of Raman backscattering by the Si substrate is systematically weaker when detected from the brighter areas. Moreover, somewhat surprisingly, the intensity difference grows with the deposited thickness of STO that the incoming and outgoing laser light need to surpass. We believe this is because of the interface layer being formed at the expense of pure Si. Second, we have occasionally seen a pair of satellite peaks around the main Si peak at ∼300 cm^–1^ (marked with arrows). These satellite peaks were observed exclusively in the bright regions. We noticed that frequencies of these satellite bands correspond well to the Raman modes of TiSi_2_.^49−51^ Since the formation of titanium silicide was revealed by TEM investigations to be more pronounced in the areas of bare Si, we thus conclude that the darker areas are those covered by rGO and the bright areas are those without rGO.

To support these observations, also 90 nm thick STO layers on an ∼50% rGO-covered surface and on a bare SiO_2_/Si surface were grown (Figure S9, SI). Raman spectra of a 90 nm thick STO layer grown on a bare Si substrate have satellite peaks at any place of the sample. It confirms the idea that these “satellites” in STO films deposited on 50% of rGO-covered surfaces come from areas without rGO. In the case of an ∼50% rGO-covered surface sample, the Raman spectrum shows that the satellite peaks were even more prominent compared to the film with a 30 nm thick film of STO, confirming thus the trend expected from Figure 4. Furthermore, we have employed the technique of Raman mapping to visually demonstrate that there is really a strong correlation between the geometry of the pattern of the optical image and the corresponding pattern of the Raman intensity map of the Si band near 300 cm ^–1^, recorded in the very same region of the sample.

Observations made on other samples with the full coverage by rGO lead us to the conjecture that in the case of the monolayer rGO coverage, the STO deposition, although performed in a vacuum of 10^–7^ mbar, may create conditions for the gradual disintegration of rGO. However, the preservation of rGO after the STO growth, in the case of bi-/three-layer coverage, may suggest that the process of disintegration is limited to a single, hence sacrificial, rGO layer.

### 3.6 Is Being Perfect Really Necessary?

**Figure 5:**
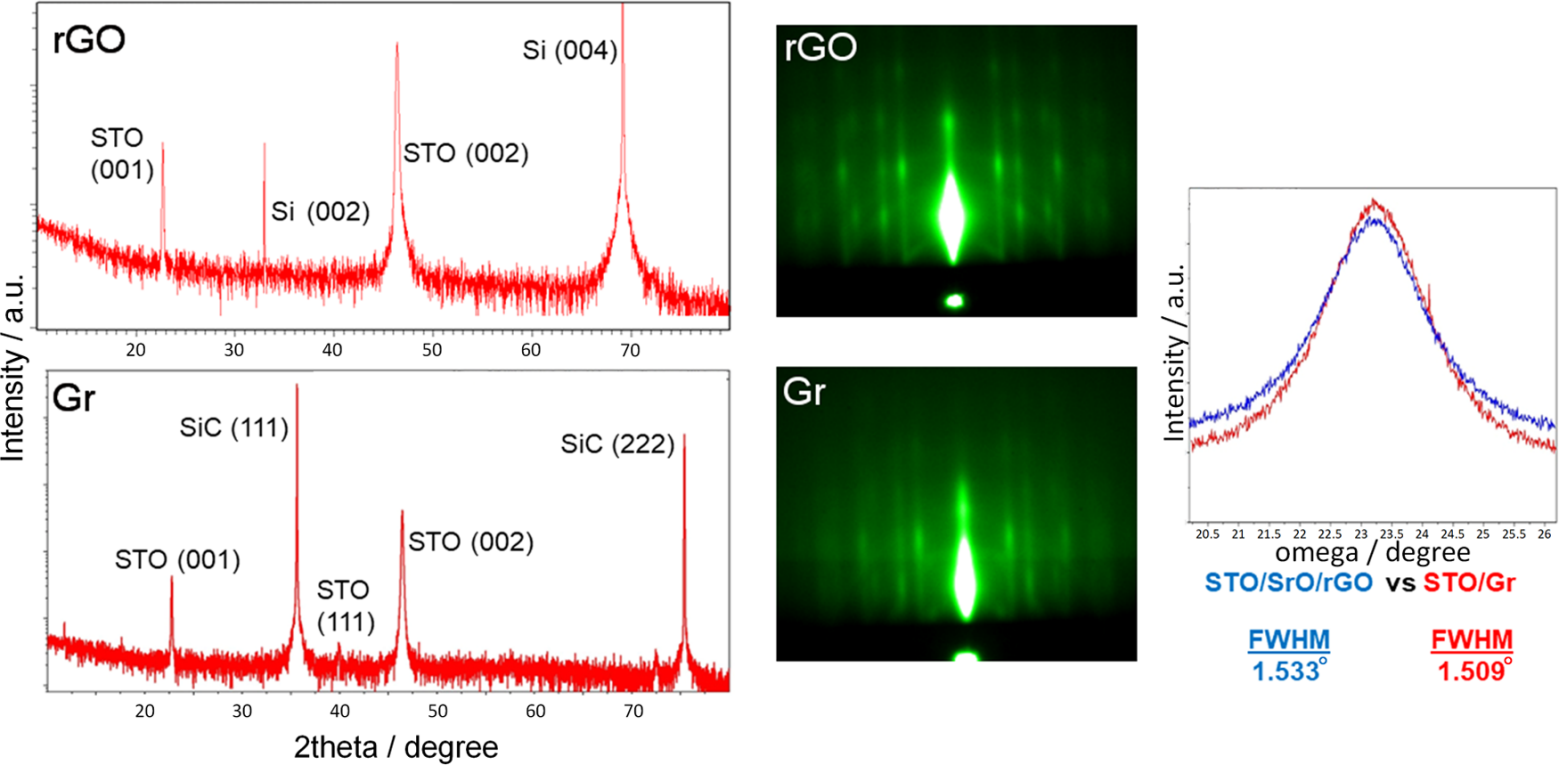
XRD, RHEED, and rocking curve analysis of a 90 nm thick STO grown on rGO and SiC-derived graphene (Gr).

One of the main advantages of the application of rGO as a buffer layer for the growth of STO and potentially other pseudo-substrates is the fact that the wet^52^ or dry^27^ transfer of graphene to the support of interests can be avoided. However, the question is to what extent the crystalline quality of graphene may play a role in the growth of STO pseudo-substrate? For this purpose, we have used high-quality SiC-derived graphene and compared it to rGO (Figure 5).

It can be noticed that STO has more “epitaxial” character in the case of rGO since only one out-of-plane orientation was noticed (in the XRD of STO on Gr/SiC, small diffraction maxima of (111) can be discerned). The same conclusion of a better crystalline quality of STO/rGO can be reached based on RHEED since streaks appear sharper and the background is less diffused compared to that of STO/Gr/SiC. Finally, a slightly lower mosaicity was observed in the case of STO/Gr/SiC compared to that of rGO, which is understandable due to the partial overlap of rGO sheets. Given that the STO growth was performed under the same conditions in both cases, better STO crystallinity on SiC-derived graphene could be expected in the case when the complete procedure (including the formation of graphene on SiC) is performed at UHV conditions, i.e., without exposure to the atmospheric conditions.

### 3.7 Beyond STO: Crystal Structure, Morphology, and Properties of PZT Grown on STO Pseudo-Substrate

**Figure 6:**
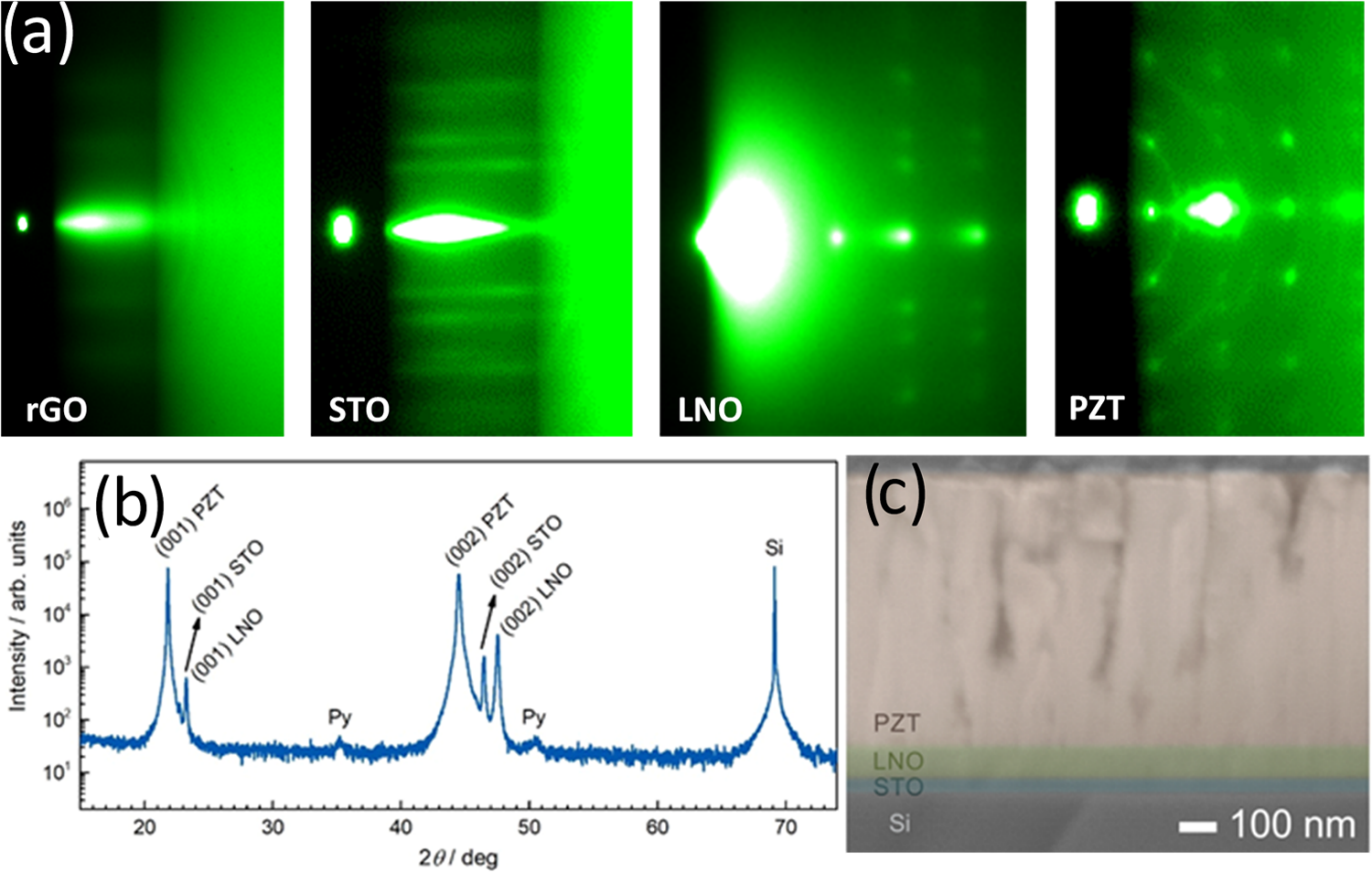
(a) Characteristic RHEED patterns of the individual layers during the PLD growth of PZT/LNO/STO on SrO(deox.)/rGO/SiO_2_/Si; (b) and (c) corresponding XRD and TEM cross section of the grown heterostructure, respectively.

In the next step, the PLD growth of PZT on STO/SrO(deox.)/rGO was considered as a way of introducing additional functionality to the silicon platform. The crystal structure of an individual layer was examined *in situ* by RHEED and *ex situ* by XRD (Figure 6). Both rGO and STO exhibited a streaky RHEED pattern, indicating a flat surface of very good crystalline quality. During the growth of the LNO layer, a transition from the layer-by-layer to the island growth mode was observed, which can be recognized by the appearance of a spotty RHEED pattern. Note that the RHEED pattern of the LNO layer was acquired at the deposition conditions (*p*(O_2_) = 0.13 mbar); therefore, the electron scattering due to background gas is enhanced. PZT continued to grow in a three-dimensional (3D) manner; therefore, the pattern is also spotty. Additionally, faint dashed rings can be observed (Figure 6, top). It is clear from the XRD (Figure 6, bottom) that the rings observed by RHEED belong to the small pyrochlore inclusions with different orientations. The concentration of the inclusions is very low (note the logarithmic intensity scale). The XRD pattern confirms that the STO, LNO, and PZT layers were grown epitaxially (in the out-of-plane direction).

The cross section of the sample reveals densely packed columnar grains in the PZT layer (Figure 6c). The low-magnification TEM image of the cross section of the entire stack reveals a columnar/granular structure that originates from the individual rGO sheets, which give rise to the formation of STO crystallites with different in-plane orientations. These columns extend across the interfaces to the LNO and PZT layers. The interface between STO and LNO is sharp and free of defects, whereas the interface between LNO and PZT is not as smooth and contains different types of defects as well as some holes (see also Figure S10, SI). The reason for this is a rougher surface (as compared to STO) of the LNO, as already recognized in the 3D RHEED pattern. Furthermore, the lattice mismatch between PZT and LNO is relatively large (4.9%), and owing to the large thickness of the PZT layer, some defects arise from the strain relaxation that occurs at the interface as the critical thickness of PZT is surpassed. As the PZT layer is cooled below the Curie temperature, the crystal symmetry is lowered and the formation of ferroelectric domains can cause further perturbances at the interface.^31^

**Figure 7:**
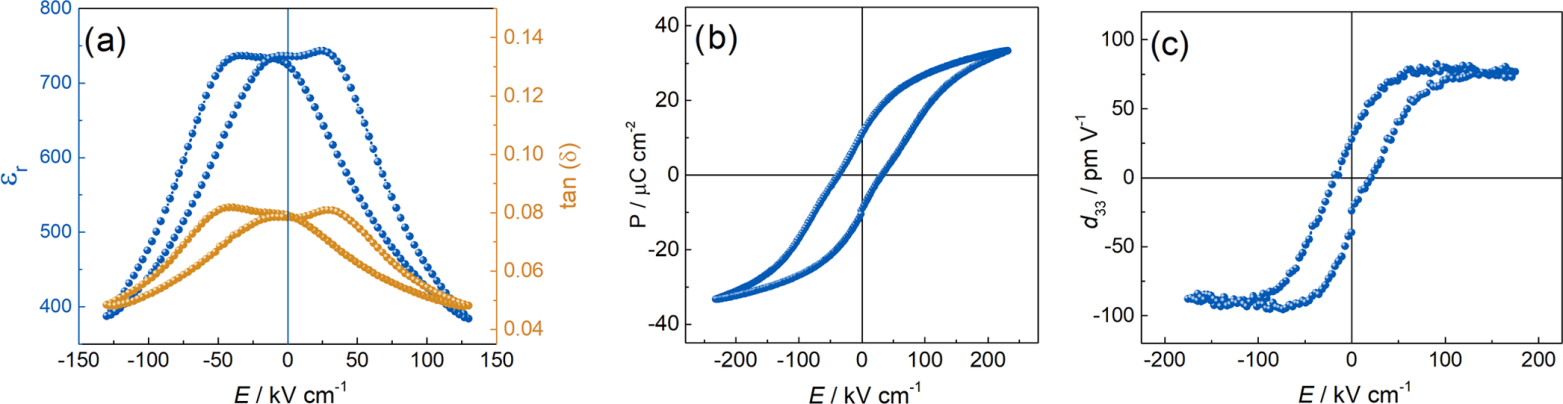
Electrical properties of the PZT layer. (a) Dielectric tunability, (b) *P*–*E* loop, and (c) piezoelectric *d*_33_ coefficient.

The electrical properties of the PZT sample are shown in Figure 7. The relative permittivity and loss tangent versus electric field hysteresis loops, exhibiting a relatively high dielectric tunability (∼50%) and low dielectric loss, are shown in panel (a). A double peak shape is observed, along with a slight pinching of the ferroelectric and piezoelectric hysteresis loops, shown in panels (b) and (c), respectively. The pinching is indicative of a small concentration of defects introduced into the film by either the deposition process or the sputtering of top Au electrodes. The value of the coercive field is *E*_c_ = ∼14 kV cm^–1^. The maximum polarization (*P*_max_ = 33 μC cm^–2^) and piezoelectric coefficient (*d*_33,max_ = 80 pm V^–1^) values are comparable to literature values for PZT thin films grown by conventional epitaxy.^34,53−56^ Note that the ferroelectric loop is not saturated due to breakdown of the film at higher electric fields.

## 4 Conclusions

Our results build upon the premises and promises of direct, van der Waals, and remote epitaxy: a synergy and integration of dissimilar systems for added functionality. In our case, the functional upgrade of the silicon platform was achieved by utilizing SrO-assisted deoxidation and controllable rGO coverage of the silicon substrate. Thanks to a tunable degree of direct and van der Waals epitaxy, both monolithic and transferable integration of dissimilar systems are prospective. Preliminary results show that STO films grown on a fully covered rGO surface can be easily delaminated because of the weak van der Waals bonds between the grown oxide films and the underlying layered substrate, thus potentially allowing growth, transfer, and stacking of numerous functional materials. Furthermore, via a simple and quick spin-coating technique, a uniform and highly reproducible rGO layer with different coverages can be easily achieved, avoiding the time-consuming graphene transfer to substrates of interest, which is very challenging and subject to a low success rate. In conjunction with the following PLD method for the growth of thin films (STO, LNO, and PZT), we demonstrate that this approach is potentially suitable for large-area device preparation. As our case study of rGO vs SiC-derived graphene shows, being perfect is not detrimental. Hence, we believe that further breakthroughs in remote epitaxy could be expected upon the seamless integration of rGO sheets with atomically defined surfaces of various substrates.

## Supporting Information Available

PLD parameters for the individual materials, AFM of different GO coverages on the SiO_2_/Si substrate, TG/DTA of GO, RHEED of STO growth on rGO-buffered silicon, RHEED of STO growth on rGO-buffered silicon after SrO deoxidation, RHEED and XRD of STO grown with different SrO deoxidation temperatures, AFM and RHEED and XRD of STO grown on bare SiO_2_/Si with and without SrO deoxidation, bright-field and STEM–EDX elemental mapping, Raman analyses, and TEM cross section of PZT/LNO/SrO(deox.)/rGO/SiO_2_/Si heterostructure (PDF)
