# Supplementary material for: Tiling the Silicon for Added Functionality: PLD Growth of Highly Crystalline STO and PZT on Graphene Oxide-Buffered Silicon Surface
Source: ACS Appl Mater Interfaces. 2023 Jan 18;15(4):6058–68. doi: 10.1021/acsami.2c17351 (PMC9906728; doi:10.1021/acsami.2c17351)
Supplement: Supplementary file 1 — am2c17351_si_001.pdf [file am2c17351_si_001.pdf]

Supporting Information

Tiling the silicon for added functionality: PLD growth of highly-crystalline STO and PZT on graphene oxide-buffered silicon surface

Zoran Jovanović,[†,‡,] Urška Trstenjak,[†] Hsin-Chia Ho,[†] Olena Butsyk,[§] Binbin Chen,[∥,⊥] Elena Tchernychova,[▽] Fedir Borodavka,[§] Gertjan Koster,[∥] Jiří Hlinka[§] and Matjaž Spreitzer[†,*]*

[†]*Advanced Materials Department, Jožef Stefan Institute, 1000 Ljubljana, Slovenia*

[‡]*Laboratory of Physics, Vinča Institute of Nuclear Sciences - National Institute of the Republic of Serbia, University of Belgrade, 11351 Belgrade, Serbia*

[§]*Department of Dielectrics, Institute of Physics of the Czech Academy of Sciences, 182 00 Prague, Czech Republic*

[∥]*MESA+ Institute for Nanotechnology, University of Twente, 7522 NB Enschede, The Netherlands*

[⊥]*Key Laboratory of Polar Materials and Devices (MOE) and Department of Electronics, East China Normal University, 200241 Shanghai, China*

[▽]*National Institute of Chemistry, 1000 Ljubljana, Slovenia*

AUTHOR INFORMATION

Corresponding Authors

* E-mails: zjovanovic@vinca.rs; matjaz.spreitzer@ijs.si

Table S1. PLD parameters for the individual materials.

Material	T [°C]	*P* [mbar]	Fluence [J cm^{-2}]	ν [Hz]	Spot size [mm^2]	Target-to-substrate distance [mm]	Number of pulses or thickness
SrO	600	8×10^{-8}	2	0.1	2.3	55	15 pulses
STO	700	1×10^{-7}	2	3	2.3	55	3 – 90 nm
LNO	570	0.13	2.5	5	2.3	55	80 nm
PZT	570	0.13	2.5	9	2.3	55	700 nm

Deposition rates:

- SrO; 0.035 nm per pulse.
- STO; 0.03 nm per pulse
- LNO; 0.015 nm per pulse
- PZT; 0.02 nm per pulse

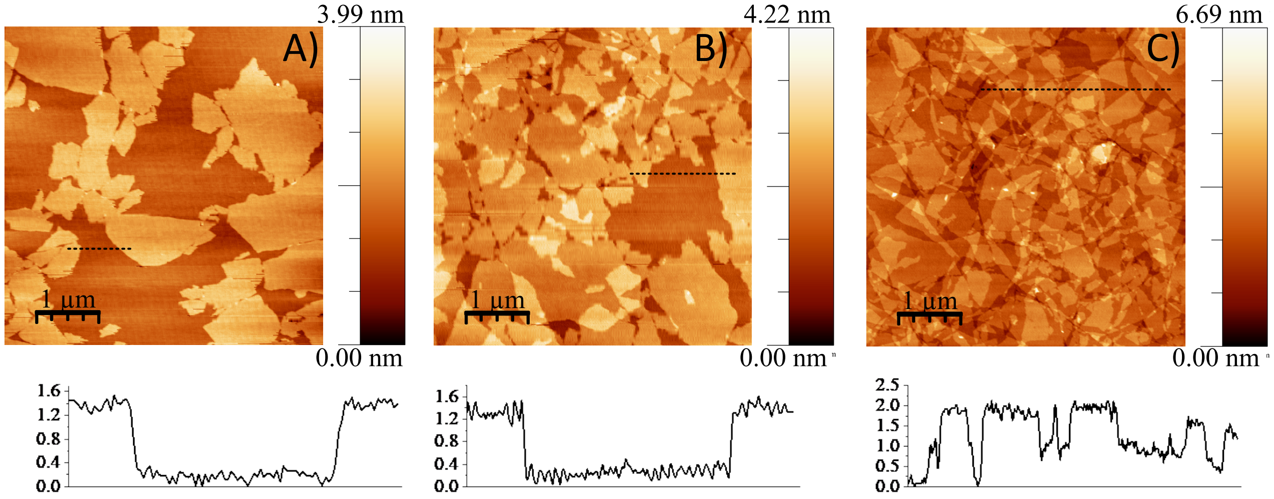

Figure S1. AFM images and line profiles of GO sheets on SiO_2/Si substrate at different coverages: **A)** ~50%, **B)** ~80% and **C)** ~100%. The AFM measurements were performed on freshly spin-coated samples, prior to thermal treatment in the PLD chamber.

The **Figure S1** shows the evolution of surface morphology of SiO_2/Si substrate with different coverage of GO nanosheets, as seen by atomic force microscopy (AFM, Veeco Dimension 3100 SPM) under tapping mode using silicon probes (OTESPA-R3, Bruker). At around 50% coverage of the SiO_2/Si surface a rather uniform distribution can be noticed, without overlapping of the GO nanosheets (**Figure**

S1A). It has to be pointed out that the piranha solution can readily modify Si surface by formation of hydroxyl groups, which fundamentally transforms the initial hydrophobic Si surface into hydrophilic. This hydrophilicity enabled easier deposition, where the basal plane of GO can strongly anchor to the hydroxylated Si surface. The lower panel of **Figure S1A** shows the line-scan profile across GO layer and bare SiO_2/Si surface, from which the estimated step-height of individual GO nanosheet was 1.0 – 1.2 nm. The presence of functional groups, structural imperfections and adsorbed/trapped water below GO nanosheet can lead to an increased step height which is in good agreement with the values reported for the GO monolayer.[1-3] By increasing the volume of the applied GO suspension during the spin-coating process, the surface coverage could be easily increased to ~ 80 % (**Figure S1B**). The corresponding height profile again clearly indicated the presence of GO sheet with thickness of ~1.2 nm. Here not only single layer can be noticed (similar to 50 % coverage case in Figure S1A), but double and/or multiple layers can be readily appreciated. The situation is even more noticeable in the full coverage (~ 100 %, **Figure S1C**)). The gradual morphological change from half coverage to full coverage *i.e.*, from mono- to bi-/three-layer coverage was simply dictated by the hydrodynamic thinning in continuous spin-coating process. In the present study, we investigated to what extent this surface arrangement of GO nanosheets would lead to distinct changes of interatomic interaction between the Si substrate and the over-grown $SrTiO_3$ *i.e.*, its crystallinity and surface quality – hence, its applicability as a pseudo-substrate.

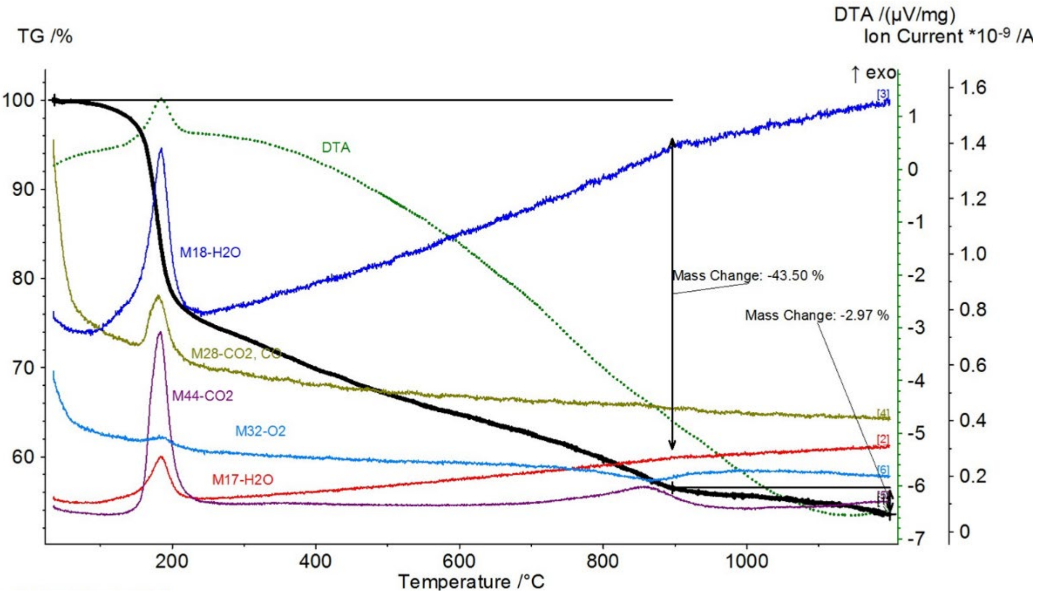

Figure S2. TG/DTA curve of bulk GO powder.

The **Figure S2** is showing that significant mass loss of the GO sample is occurring at ~200 °C, which is in line with desorption of epoxy groups from GO basal plane.[4] Next, we can appreciate a second

mass loss occurring from 200 – 900 °C, and third from ~900 °C – 1200 °C. The second mass loss might originate from desorption of oxygen functional groups of higher stability that also tend to remove part of carbon atoms from the graphene matrix itself (> 700 °C, a change of slope can be noticed). Beside this process, the reaction of rGO with SiO_2/Si support should also be taken into account, that would lead to reduction of SiO_2 *i.e.*, combustion of graphene. Although we didn't investigate this in detail, we believe that this process is ongoing both in second and third stage of mass loss. Also, at temperature range of third mass loss, a spontaneous deoxidation of bare SiO_2/Si surface is expected to occur. For example, flash annealing at ~1200 °C for less than a minute is sufficient for complete removal of native oxide from silicon surface.[5]

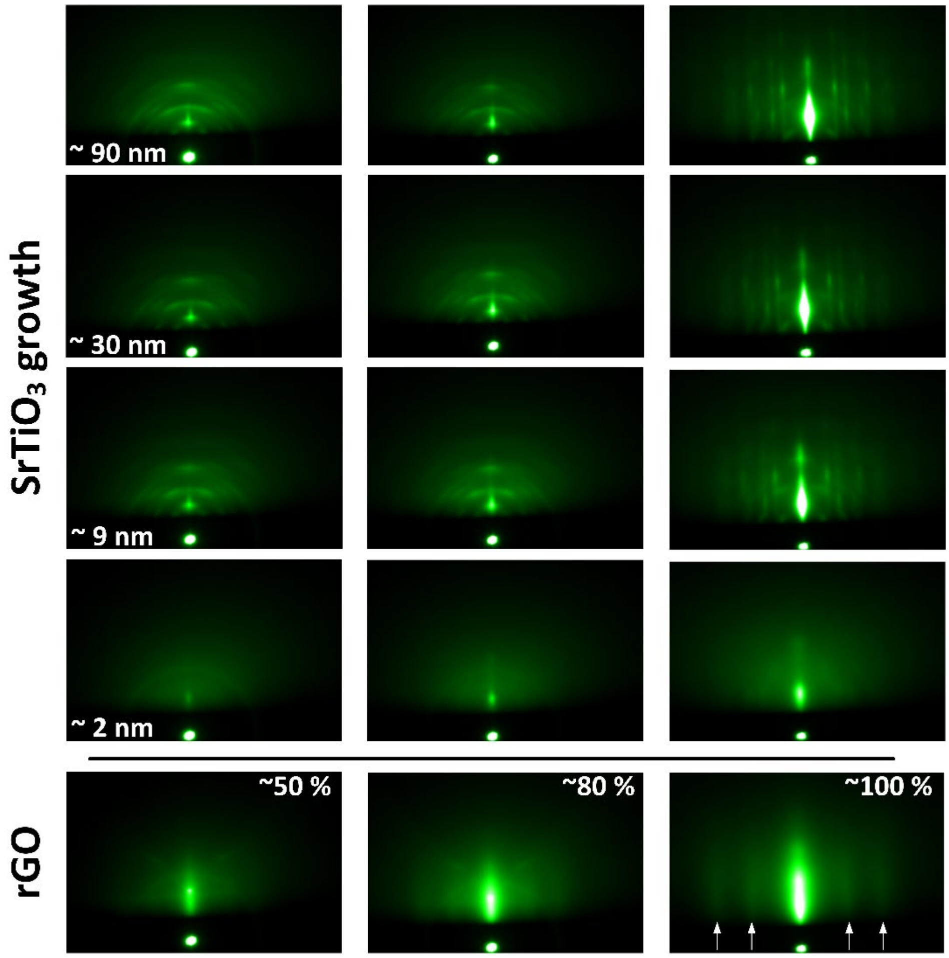

Figure S3. RHEED images of STO growth on rGO-buffered silicon surface (~ 50, ~ 80 and ~ 100 % coverage). Arrows point to streaks of graphene lattice of rGO.

The **Figure S3** shows the evolution of STO crystal structure during its growth on SiO_2/Si surface that was buffered by rGO nanosheets at characteristic coverages (~50, ~80 and ~100 %). After the samples were annealed and GO reduced to rGO, it could be seen that faint and broad streaks corresponding to

graphene lattice with certain degree of ordering appeared, which is the most visible in 100 % coverage (rGO part of Figure S3). These faint streaks are an indication of good crystalline quality of rGO,[6] presumably from restored *sp*^{2}-network due to desorption of basal-plane functional groups. As the first few pulses of STO were deposited the RHEED patterns drastically changed, where no feature associated with graphene but only specular beam was discernible (up to ~2 nm of STO). During STO deposition, between 2 and 9 nm, the RHEED patterns have essentially been established, *i.e.*, their appearance would remain the same throughout deposition process (9, 30 and 90 nm). In the case of 50 % coverage sample, the RHEED pattern features both a series of broken rings and broad/elongated spots which are characteristic of textured surface. Similarly, the RHEED pattern of 80 % rGO coverage sample shows the same features. This can be justified by the STO growth on two separate regions; one - bare SiO_2/Si substrate where plasma plume with high energy can easily lead to formation of silicates because of which epitaxial growth was difficult to achieve,[7] and the other – graphene-like regions upon which STO layer can crystallize in single orientation without surface potential interference from the underneath substrate (SiO_2/Si). The contribution by polycrystalline parts at ~50 and ~80% coverage seemed to dominate the RHEED patterns, although the signal from STO preferred orientation was more pronounced at ~80% coverage. The STO grown on full rGO coverage showed the clearest streaky RHEED patterns (among the three samples) along with minor rings, indicating good crystallinity and smoothness of the sample's surface.

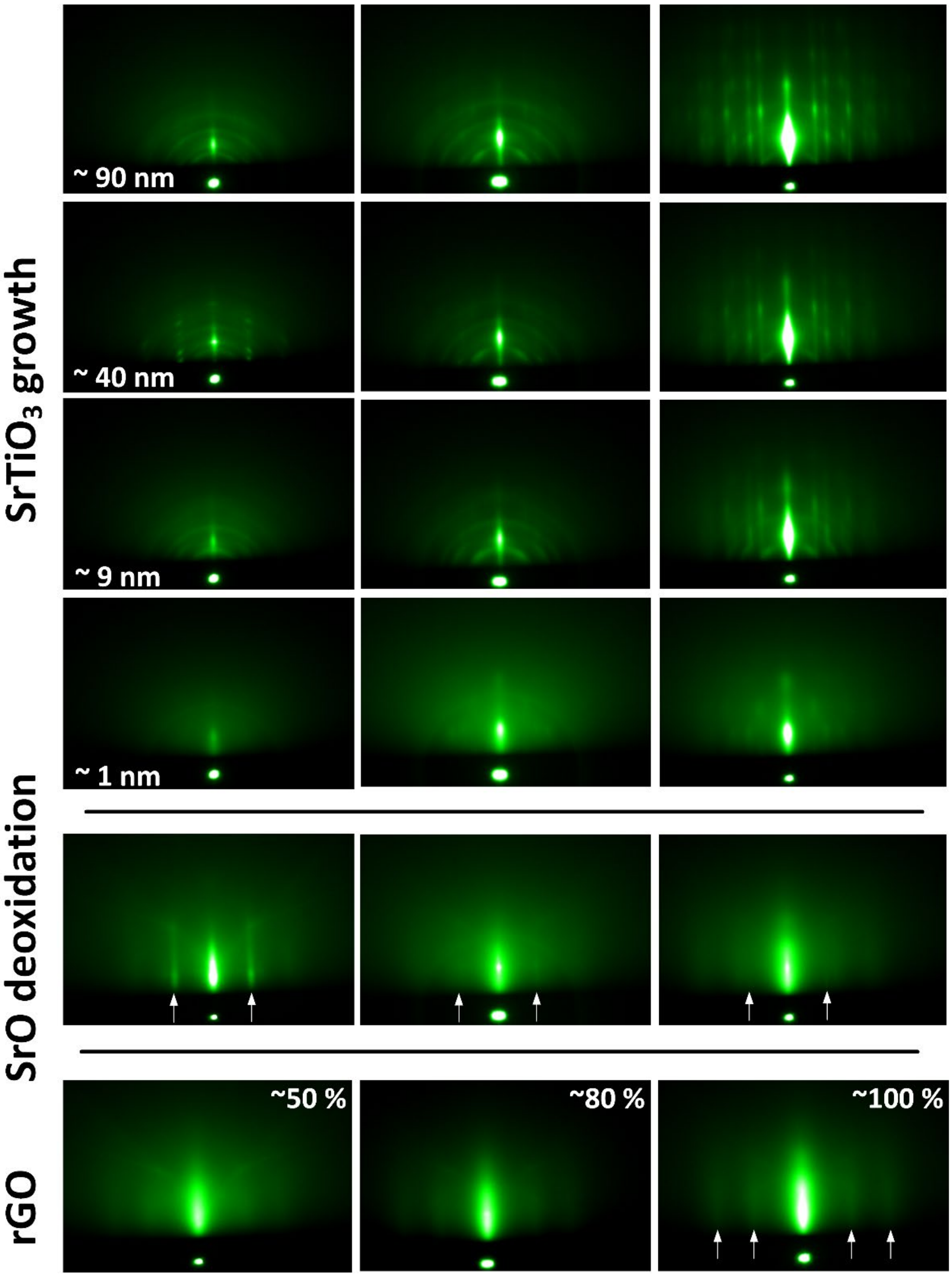

Figure S4. RHEED images of STO growth on rGO-buffered silicon surface (~ 50, ~ 80 and ~ 100 % coverage) after SrO deoxidation step. In rGO section arrows point to streaks of graphene lattice. In SrO deoxidation section arrows point to 1× streaks of strontium reconstruction of silicon surface, obtained along <100> azimuth.

The general behavior of samples prepared with the SrO-induced deoxidation and reconstruction of Si surface, in comparison to the ones fabricated by omitting SrO step, can be understood from **Figure S4**. As can be seen, after the SrO-assisted deoxidation, sharp streaks belonging to 1× Sr-reconstructed surface can be nicely seen,[5,7] indicating that in the regions of bare Si *i.e.*, without rGO, a native oxide SiO_2 is effectively removed and the silicon surface undergoes a complete reconstruction. Intensity of streaks diminishes as rGO coverage increases and finally the streaks are hardly observable in 100 % rGO coverage sample because of almost complete coverage of SiO_2/Si surface. This is additional confirmation that the streaks are originating from bare SiO_2/Si regions. Streaks representative of STO two-dimensional growth can already be appreciated at STO thickness of ~ 1 nm (~ 2 u.c.), especially in 80 and 100 % coverage samples, which is in stark contrast compared to samples prepared without SrO, where the streaks are still not discernible at thickness of ~ 2 nm (Figure S3). As the deposition proceeds, the streaky patterns become stronger in signal (100 % rGO coverage), suggesting the STO is grown in a continuous and well-defined layer-by-layer mode. On the other hand, concurrence of streaks along with broken rings in 50 % and 80 % rGO coated samples implies that in SrO-deoxidized regions an STO layer is textured. In the full coverage sample, the sharp streaks with higher intensity indicate the STO epilayer is prepared with higher quality compared to that without SrO deoxidation step, as is evidenced by the XRD results (*see Figure 1 in the main text*). Based on this, it can be confirmed that the synergistic effects of rGO and SrO deoxidation are of great importance for STO epilayer formation *i.e.*, the realization of added functionality to Si platform using the PLD technique.

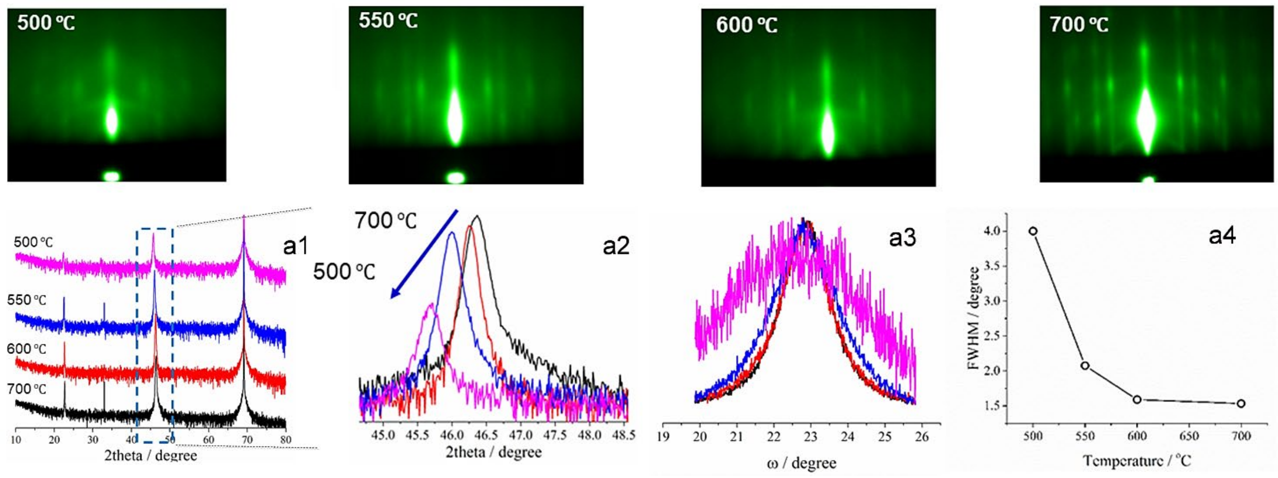

Figure S5. The RHEED patterns of 90 nm-thick STO grown on fully rGO-covered SiO_2/Si surface, including SrO-deoxidation step (500–700 °C). **a1**) The *θ-2θ* scan of the corresponding STO films; **a2**) a zoom-in to a region of STO (002) maxima; **a3)** rocking curve analysis of STO (002) diffraction maxima; **a4)** the FWHM of the obtained rocking curves of STO (002) maxima.

The **Figure S5** is showing that single out-of-plane orientation of STO (002) can be obtained also at 500 °C, but at a cost of lower crystalline quality. This is noticeable from the RHEED patterns that are becoming more diffuse as temperature decreases (**Figure S5, top segment**). Also, as a shift to lower *2θ* values of STO (002) maximum (**Figure S5a1**) and increase of FWHM of rocking curves of STO (002) diffraction maxima corroborate this observation (**Figure S5a4**). Having in mind that deposited STO will be used as a pseudo-substrate, we have preserved 700 °C as an optimal deposition temperature.

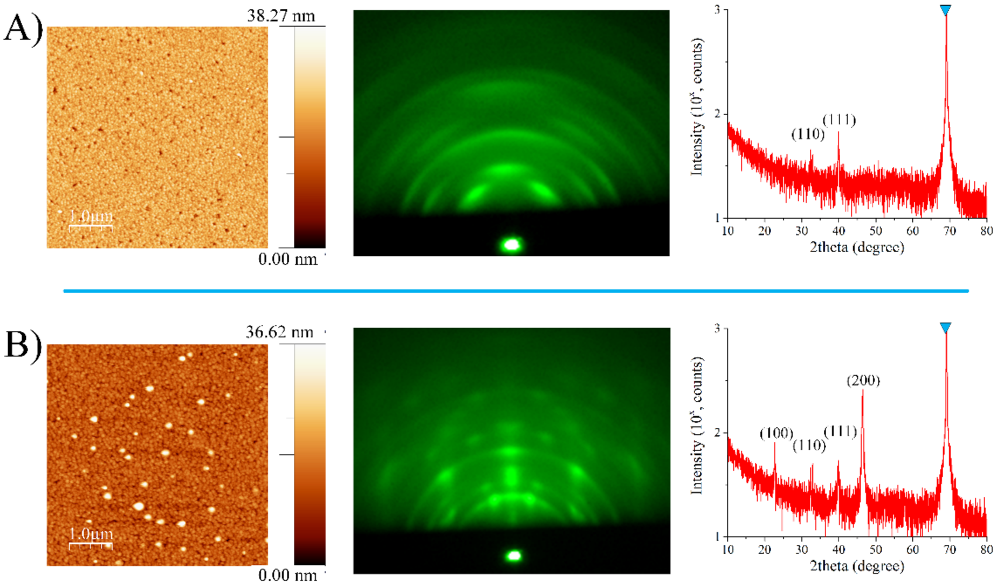

Figure S6. STO grown on bare SiO_2/Si **A)** without and **B)** with SrO deoxidation as seen by AFM (left), RHEED (middle) and XRD (right) methods.

90 nm-thick STO layer was also grown directly on bare Si substrate without SrO-assisted deoxidation. As viewed from the surface morphology (**Figure S6A, left**), the STO surface is homogeneous with the estimated root mean square (RMS) value of ~3 nm, whereas substantial pinholes can be clearly observed, implying that the reaction between the film and substrate results in the formation of structural defects, which ultimately degrade the film quality. The acquired RHEED image exhibits a series of broken rings (**Figure S6A, middle**), revealing the polycrystalline feature with textured surface. Moreover, the diffuse background can be clearly observed, which is characteristic of amorphous film. The relatively low intensities of STO (011) and (111) orientations in XRD result (**Figure S6A, right**) again indicate growth of STO of poor crystalline quality.

In another sample, optimal procedure for SrO-deoxidation was used prior to STO layer deposition. The surface of STO film is quite rough with RMS of ~3.6 nm owing to numerous large particles formed on the STO surface (**Figure S6B, left**). Spotty patterns together with rings are clearly evidenced in RHEED image as shown in the middle of **Figure S6B**. Polycrystalline feature in parallel with preferred (002) orientation can be seen in XRD, as shown in **Figure S6B, right**. One can easily notice the substantial improvement in terms of STO crystallinity when SrO-assisted deoxidation was used compared to the sample where STO is directly deposited on SiO_2/Si substrate.

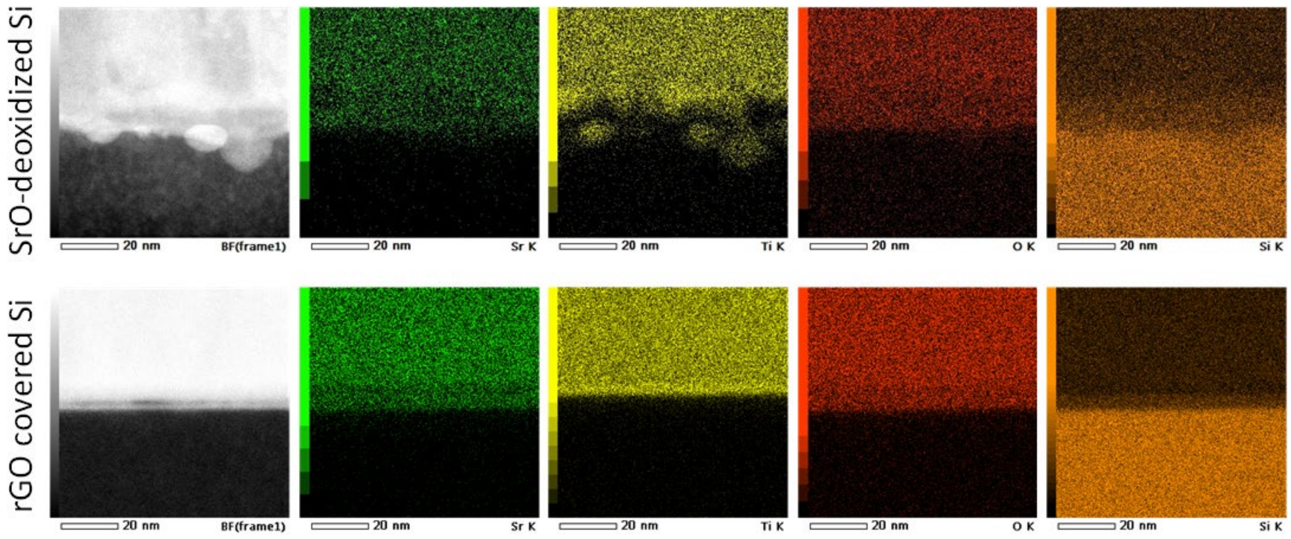

Figure S7. The bright field and STEM-EDX elemental mapping (Sr, Ti, O and Si) of the interface after the growth of ~85 nm-thick STO layer on SrO-deoxidized and rGO-covered silicon surface (top and bottom segments, respectively).

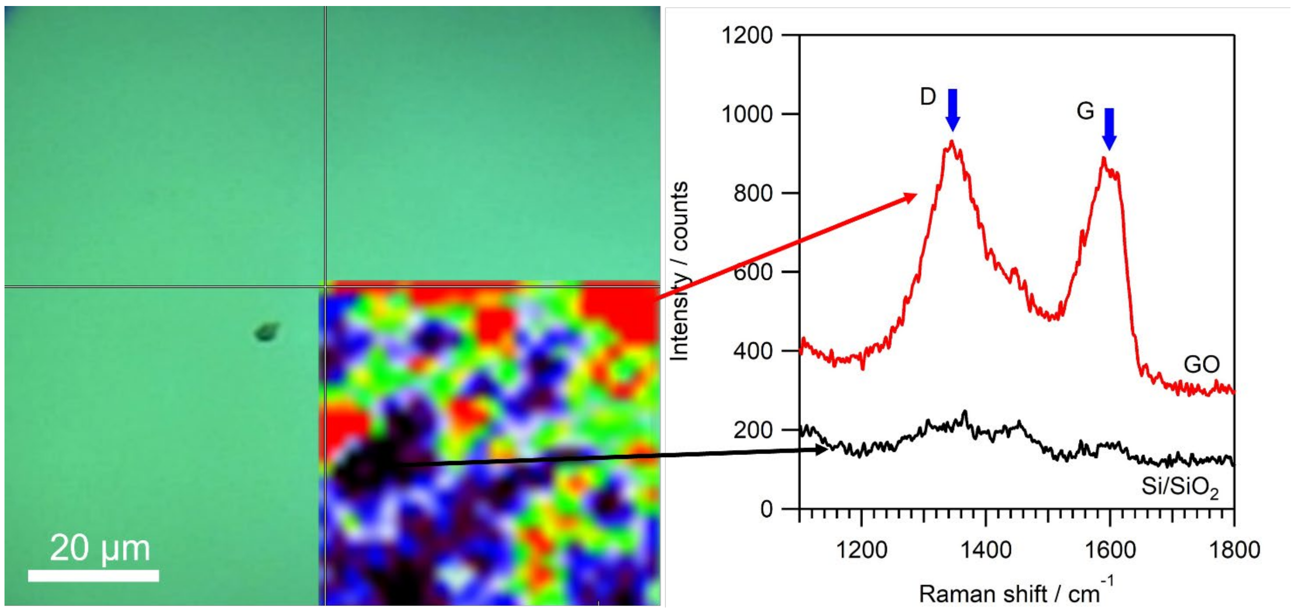

Figure S8. Raman analysis of GO-buffered SiO_2/Si surface (~50% coverage). An image by the optical microscope is shown, with in intensity map as inset (at 1603 cm^{-1}). It can be concluded that the red-colored regions are from GO and blue/black are from bare SiO_2/Si surface.

The **Figure S8** shows the Raman spectra of D and G bands of GO nanosheets deposited on the SiO_2/Si surface taken before the follow up thermal treatment and STO deposition was performed. In this case the Raman mapping of the intensity of the G band can be conveniently used to detect the areas covered by GO nanosheets and without it and verify the GO quality. The red-colored regions in the Raman

intensity map indicate presence of GO, and blue/black are from the bare SiO_2/Si surface. Due to the finite spatial resolution of the techniques, some residual intensity at D and G bands is probably leaking also to the solid black spectrum in the inset which otherwise should mainly show residual scattering by SiO_2/Si substrate. In particular, the broad band near 1450 cm^{-1} may be attributed to third order TO phonon mode (3TO) from Si substrate.[8]

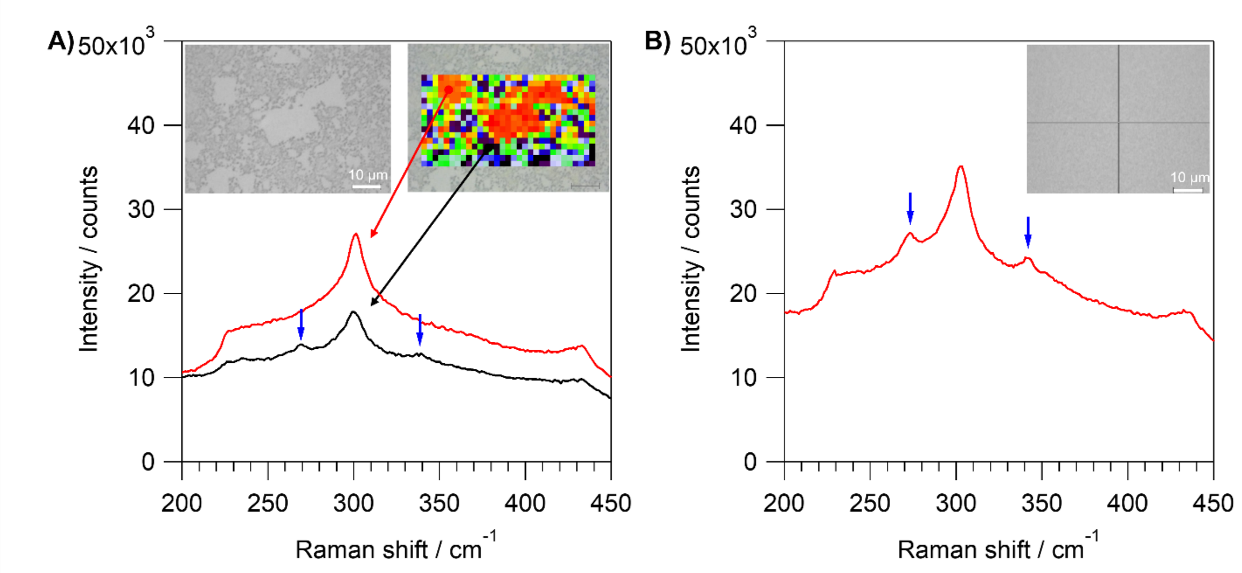

Figure S9. Raman analysis of 90 nm thick STO layer grown on **A)** rGO-buffered SiO_2/Si surface (~50% coverage) and on **B)** a bare SiO_2/Si surface. Arrows are pointing to the “satellite” peaks that were noticed only on bare Si surface. In top left and right corners of **A)** an optical image and intensity map (at 300 cm^{-1}) are shown, respectively; areas of rGO-covered surface appear brighter in an optical image of this sample (it has an opposite contrast to the samples shown in Figure 4 in the main text).

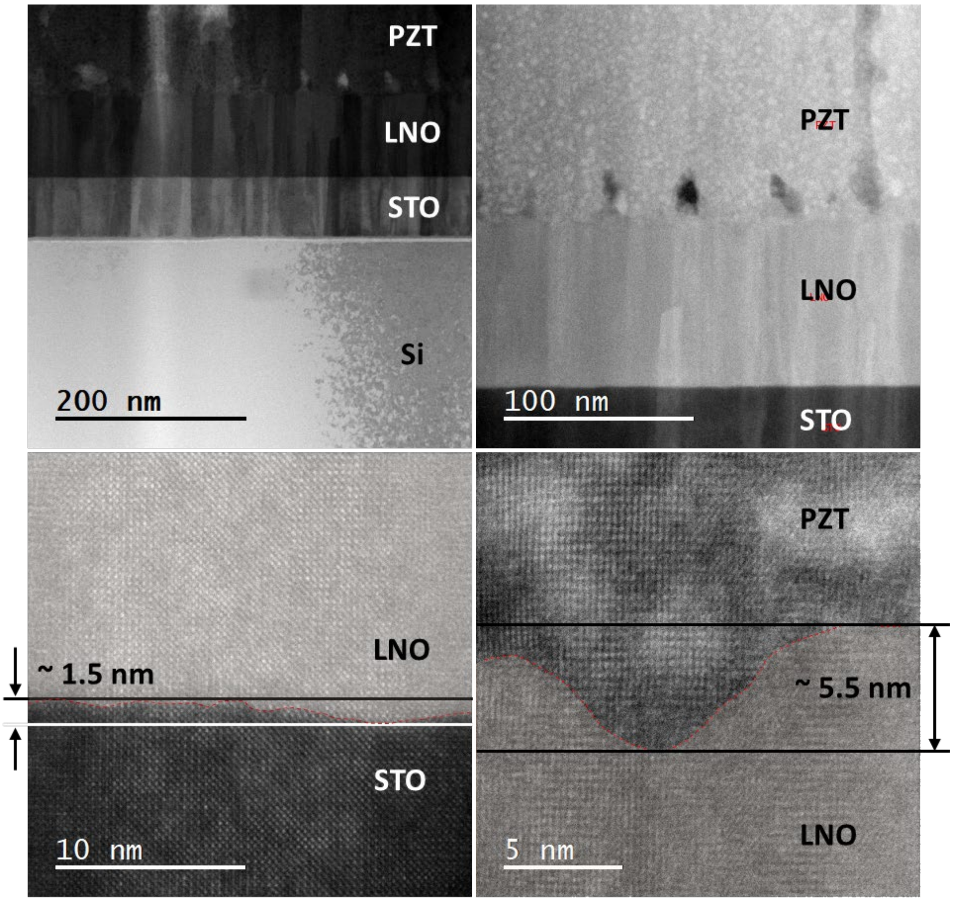

Figure S10. TEM cross-section of the PZT/LNO/STO/SrO(deox.)/rGO/SiO_2/Si heterostructure. The STO/LNO interface is smoother compared to the LNO/PZT interface (≤ 3 nm *vs.* ≤ 7 nm, respectively, across the whole sample).

References:

1. Eda, Goki, Giovanni Fanchini, and Manish Chhowalla. "Large-area ultrathin films of reduced graphene oxide as a transparent and flexible electronic material." Nature nanotechnology 3.5 (2008): 270-274.

2. Marcano, Daniela C., et al. "Improved synthesis of graphene oxide." ACS nano 4.8 (2010): 4806-4814.

3. Pei, Songfeng, and Hui-Ming Cheng. "The reduction of graphene oxide." Carbon 50.9 (2012): 3210-3228.

4. Jovanovic, Z., et al. "The role of surface chemistry in the charge storage properties of graphene oxide." Electrochimica Acta 258 (2017): 1228-1243.

5. Jovanović, Zoran, et al. "Control of SrO buffer-layer formation on Si (001) using the pulsed-laser deposition technique." RSC advances 6.85 (2016): 82150-82156.

6. Lu, Zonghuan, et al. "Revealing the crystalline integrity of wafer-scale graphene on SiO_2/Si: an azimuthal RHEED approach." ACS Applied Materials & Interfaces 9.27 (2017): 23081-23091.

7. Jovanović, Zoran, et al. "Silicon surface deoxidation using strontium oxide deposited with the pulsed laser deposition technique." ACS Applied Materials & Interfaces 6.20 (2014): 18205-18214.

8. Cao, Peigen, et al. "The microscopic structure of adsorbed water on hydrophobic surfaces under ambient conditions." Nano letters 11.12 (2011): 5581-5586.
